# Creating and Validating an Algorithm to Measure AIDS Mortality in the Adult Population using Verbal Autopsy

**DOI:** 10.1371/journal.pmed.0030312

**Published:** 2006-08-08

**Authors:** Ben A Lopman, Ruanne V Barnabas, J. Ties Boerma, Godwin Chawira, Kezia Gaitskell, Tara Harrop, Peter Mason, Christl A Donnelly, Geoff P Garnett, Constance Nyamukapa, Simon Gregson

**Affiliations:** 1 Department of Infectious Disease Epidemiology, Imperial College London, London, United Kingdom; 2 Somerville College, Oxford University, Oxford, United Kingdom; 3 Measurement and Health Information Systems, Evidence and Information for Policy, World Health Organization, Geneva, Switzerland; 4 Biomedical Research and Training Institute, Harare, Zimbabwe; Rosetta Genomics, Israel

## Abstract

**Background:**

Vital registration and cause of death reporting is incomplete in the countries in which the HIV epidemic is most severe. A reliable tool that is independent of HIV status is needed for measuring the frequency of AIDS deaths and ultimately the impact of antiretroviral therapy on mortality.

**Methods and Findings:**

A verbal autopsy questionnaire was administered to caregivers of 381 adults of known HIV status who died between 1998 and 2003 in Manicaland, eastern Zimbabwe. Individuals who were HIV positive and did not die in an accident or during childbirth (74%; *n* = 282) were considered to have died of AIDS in the gold standard. Verbal autopsies were randomly allocated to a training dataset (*n* = 279) to generate classification criteria or a test dataset (*n* = 102) to verify criteria. A rule-based algorithm created to minimise false positives had a specificity of 66% and a sensitivity of 76%. Eight predictors (weight loss, wasting, jaundice, herpes zoster, presence of abscesses or sores, oral candidiasis, acute respiratory tract infections, and vaginal tumours) were included in the algorithm. In the test dataset of verbal autopsies, 69% of deaths were correctly classified as AIDS/non-AIDS, and it was not necessary to invoke a differential diagnosis of tuberculosis. Presence of any one of these criteria gave a post-test probability of AIDS death of 0.84.

**Conclusions:**

Analysis of verbal autopsy data in this rural Zimbabwean population revealed a distinct pattern of signs and symptoms associated with AIDS mortality. Using these signs and symptoms, demographic surveillance data on AIDS deaths may allow for the estimation of AIDS mortality and even HIV prevalence.

## Abstract 2

Based on verbal autopsy from patients in rural Zimbabwe, 69% of deaths were correctly classified as AIDS/non-AIDS. The authors say the method can be used in demographic surveillance on AIDS deaths.

## Editors' Summary

**Background.**

The worldwide epidemic of HIV/AIDS is at its most severe in poor countries—for example, in Africa, where over 25 million people are estimated to have been infected with HIV. Such countries generally lack the kind of health system that is able to record the cause of death for each person who dies. This makes it very hard to know the number of people whose deaths are related to AIDS. The uncertainty over the AIDS death rates in these countries causes many problems. For example, it will be hard to establish how effective the introduction of AIDS treatment drugs is proving to be in reducing the number of AIDS deaths. A new approach is needed to determine and record the causes of deaths in these countries. It is not enough to rely on knowing whether the person who has died has been found to be HIV positive; many people in poor countries who have AIDS have never been tested, and, in any case, the cause of death in a person with HIV may not necessarily be related to the virus.

**Why Was This Study Done?**

The researchers wanted to investigate the use of the technique called “verbal autopsy.” This involves asking those who were with the deceased person before they died—usually close family members—a series of standard questions about the symptoms the person had. This is now a recognized technique, but the researchers wanted to know specifically how effective it can be in identifying AIDS as the cause of death. It is also important to “validate” the set of questions chosen to be used in a verbal autopsy, to see how likely they are to produce the correct explanation for the cause of death.

**What Did the Researchers Do and Find?**

The researchers drew up a list of what they considered appropriate questions and used them in interviews (conducted by nurses) with the caregivers of 381 adults who died between 1998 and 2003 in Manicaland, a rural area of eastern Zimbabwe. For all of these people, it was known whether they were HIV positive or HIV negative. (This information is not known for most deaths in rural Zimbabwe.) The 282 people who were HIV positive and did not die in an accident or during childbirth were considered by the researchers to have died of AIDS. (They argue this was a reasonable assumption to make, based on what is already known about death rates in HIV-positive and HIV-negative people in Zimbabwe.)

The questions the caregivers were asked about the condition of the person who died included whether or not they had any of the following signs or symptoms that are common among patients with AIDS: moderate or severe weight loss, jaundice, herpes zoster, presence of abscesses or sores, oral candidiasis, acute respiratory tract infections, and vaginal tumours. Explanations of what these conditions looked like were given to the caregivers. There were also questions on the circumstances surrounding each death. Using the answers to the questions, it was possible in 69% of cases to make a correct decision as to whether the death was the result of AIDS.

**What Do These Findings Mean?**

A verbal autopsy using the signs and symptoms selected by these authors can be used to make reasonable estimates of the number of AIDS deaths. The authors believe that, with the same set of questions, it might also be possible to produce estimates of the number of people living with HIV/AIDS. Validation of the questions used should, however, be carried out separately in each country that adopts this technique.

**Additional Information.**

Please access these Web sites via the online version of this summary at http://dx.doi.org/10.1371/journal.pmed.0030312.

• MedlinePlus has many pages providing information about HIV/AIDS.

• Information about the global epidemic of HIV/AIDS and international efforts to fight it can be found on the Web site of the Joint United Nations Programme on HIV/AIDS (UNAIDS).

• Of particular relevance is UNAIDS' country-by-country analysis of the situation in Africa.

• The World Health Organization's pages on HIV/AIDS. A recent discussion on verbal autopsies has been published by WHO.

• The Manicaland HIV/STD Prevention Study.

## Introduction

UNAIDS estimates that 24.5 million people in sub-Saharan Africa were living with HIV by 2005. The estimated annual 2.0 million AIDS-related deaths in sub-Saharan Africa in 2005 represent a dramatic impact on patterns of adult mortality [1]. However, direct assessment of the level and causes of mortality is not possible in the majority of high-mortality countries, because vital registration and cause of death reporting is incomplete or absent [2,3].

The demand for accurate data on levels and trends in AIDS mortality is increasing. Mortality statistics are an important advocacy tool and, in the era of antiretroviral therapy (ARV), the documentation of trends in AIDS mortality becomes even more important, as has been shown in the developed countries [4,5]. AIDS mortality in the population needs to be measured in order to evaluate the effectiveness of ARV scale-up as well as programmes to treat opportunistic infections [6].

In the absence of vital registration, data on the levels and trends of AIDS mortality in the general population are derived from health facilities, special population-based studies (e.g., demographic surveillance systems). Mortuary studies [9,10] and burial record surveillance [11] are additional sources of AIDS mortality that have been used in some countries. Mortality records from hospitals are generally not a reliable indicator of population mortality trends by cause because of low utilization and the likelihood of changes in utilization patterns over time. Clinical cohorts of people living with HIV/AIDS are adequate to assess the impact of survivorship in enrolled patients, but the effects in the wider community may differ due to poor adherence to regimes, inequitable access to ARV, and/or bias in selection of clinical cohorts. Therefore, tools are required urgently that will adequately assess the impact of ARV drugs in the population; in this context an improved means for ascertaining AIDS deaths is essential [7].

Demographic surveillance systems provide valuable information on trends in age- and sex-specific mortality [8], and may also include interviews with the relatives of the deceased—referred to as verbal autopsy—to ascertain a probable cause of death. HIV serostatus of the deceased individual is, however, often unavailable, and a reliable tool, with known sensitivity and specificity, would allow estimation of the level of AIDS mortality in populations with unknown serostatus.

The World Health Organisation has recognised the value of the verbal autopsy (VA) in settings lacking vital registration, and has called for validation studies for the VA tool in general, as well as for specific causes [7]. Validation studies typically compare assigned cause of death from VAs to hospital records. However, hospital records may be a poor reference standard for AIDS in communities where the deceased and their caregivers are often unaware of HIV status, a social stigma surrounds AIDS [12], hospital utilization is limited, treatment sources are traditional, or home-based care is widely used. The fact that contact with the health services is the inclusion criteria for the validation study is likely to have a major effect on the results.

The Manicaland HIV/STD Prevention Project eastern Zimbabwe, where the HIV serostatus of individuals is known prior to death, provides a unique opportunity to identify the signs and symptoms differentially associated with AIDS deaths in the pre-ARV era. Verbal autopsy data along with HIV status are used to explore a method of creating a diagnostic algorithm and assessing its performance.

## Methods

### Study Population, Mortality Surveillance, and Administration of the Verbal Autopsy Questionnaire

The Manicaland Project includes a population-based open cohort study of four subsistence farming areas, two roadside trading centres, four forestry, tea and coffee estates, and two small towns in the rural province of Manicaland in eastern Zimbabwe [13]. A baseline survey took place from 1998 to 2000, with follow-up occurring three years later in each site. Testing for presence of HIV antibody at baseline was performed on dried blood spots [14]. HIV prevalence was 15% for males (aged 17–44 y) and 21% for females (15–44 y) at baseline [15]. Male (17–54 y) and female (15–44 y) local residents in a baseline household census were considered eligible for the study. The study team identified deaths through the use of checklists of all individuals interviewed at baseline and discussions with village health workers, employers, and surviving household members present at follow-up. When a death was ascertained, a nurse conducted an interview using a structured verbal autopsy questionnaire with the primary caregiver prior to death. If that person was unavailable, a kin, a neighbour, or community health worker was interviewed. Based on the frequency of missing responses, primary caregivers (*n* = 180) did not provide more complete interviews than other respondents (*n* = 201). Data were collected on the signs, symptoms, and circumstances preceding death using a structured, closed, interviewer-led questionnaire (Protocol S1). The verbal autopsy questionnaire was based on one previously developed in Tanzania [16]. The questionnaire was translated into Shona, the predominant local language, and back-translated into English. Interviewers were certified nurses who were trained by a lead nurse in how to administer the verbal autopsy questionnaire. Data were entered into an SPSS for DOS database.

### Analysis

#### Gold standard of AIDS deaths.

For the purposes of the gold standard, we defined an AIDS death as an individual who was (a) HIV positive at baseline survey based on antibody testing and (b) was not reported to have suffered major injury from motor vehicle accident, injury that was self-inflicted (suicide), or that was accidentally (accident) or deliberately inflicted by another person (homicide) in the two weeks prior to death. For females, direct obstetric deaths—defined as (a) death shortly before delivery, with excessive bleeding and/or severe headaches, or (b) death during childbirth—were classified as being non-HIV-associated. This categorisation scheme is justified because (a) mortality rates in HIV-positive individuals are nine and ten times higher in males and females, respectively, in this population [17] and (b) preliminary analyses demonstrated that childbirth-associated deaths were proportionally more common in deaths amongst HIV-negative women (6%; *n* = 3) than those of HIV-positive women (2%, *n* = 3), as has been observed in other studies [9]. Misclassification in the gold standard was estimated by applying the underlying hazard of death (mortality rate in HIV-negative population) to the HIV-positive population, and then subtracting the “observable” misclassifications from obstetric and accident/injury deaths. Given the high sensitivity and specificity of the HIV-1 antibody diagnostic dipstick [14] and the very low probably of AIDS death occurring within three years of infection, we assumed no misclassification of individuals who were HIV negative at baseline.

#### Train and test datasets.

Two datasets were created from all deaths for which full information was available for analysis. The first dataset (train) was used to create the diagnostic algorithm, as described below. The algorithm was then applied to the second dataset (test), in order to evaluate how accurately deaths were classified. Each case had an independent chance (0.75:0.25) of being assigned to the train or test dataset. A random number ranging from 0 to 1.0 was generated for each case; cases with a number 0.75 or lower were assigned to the train dataset

#### Construction of the “diagnostic” algorithm.

The strength of association of a range of clinically relevant signs and symptoms with the outcome of AIDS death was measured by calculation of the likelihood ratio (LR), where LR = sensitivity / (1 – specificity). Each sign and/or symptom with a LR of 1.92 (corresponding to a significant χ^2^ test on 1 degree of freedom at *p* < 0.05) or greater was retained for further analysis. The focus of the algorithm was on specificity because (a) we knew a priori that most adult deaths were HIV-associated and (b) we wanted to err conservatively by avoiding false positive results. The classification algorithm was then constructed with signs/symptoms added stepwise in an iterative process. First, the sign/symptom with the highest specificity for AIDS deaths was identified. All deaths with this symptom present were then classified as AIDS-associated. The specificity of all the remaining signs/symptoms were then recalculated using only those cases not classified as AIDS-associated based on a previously included sign/symptom. This procedure was repeated until all criteria were added stepwise.

#### Analysis of the diagnostic algorithm.

In order to visualise the trade-off between increasing sensitivity and decreasing specificity with each additional sign/symptom, modified receiver operator characteristic (ROC) curves were plotted. (The plots are “modified” in that traditional ROCs display different cut-off points of a single parameter in the same model: here we are using a “lower cut-off” by adding less-specific criteria to the algorithm.) The best cut-off value from the modified ROC curve was selected by choosing the point closest to the upper left hand-corner of the plot.

In order to assess the reliability of the classification scheme, we examined associations between characteristics of the verbal autopsy procedure, the respondents, or the deceased, and the proportion correctly classified. Logistic regression models were fitted using “correctly classified” (Y/N) as the dependent variable.

#### Post-test probability.

In this study, post-test probability (+) was the likelihood that an individual death was due to AIDS (according to the gold standard) after the verbal autopsy algorithm provided a positive result—that is, AIDS death. The post-test probability was calculated with the following formula:

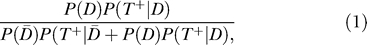
where *P(T^+^|D)* is the probability of a positive test amongst AIDS deaths; 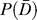 is the probability of non-AIDS death; and 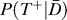 is the probability of a positive test amongst non-AIDS deaths. For this analysis, the pre-test probability, *P(D),* that an individual died of AIDS was set at 0.65—the population attributable fraction of 17- to 44-year-old mortality attributable to HIV, where the population attributable fraction represents the proportion of all deaths that would be averted if AIDS mortality was removed completely [17]. The post-test probability was then updated based on the participant meeting the criteria or not.

## Results

### HIV-Positive and -Negative Deaths, Randomisation, and Classification Based on Gold Standard Criteria

**Figure 1:**
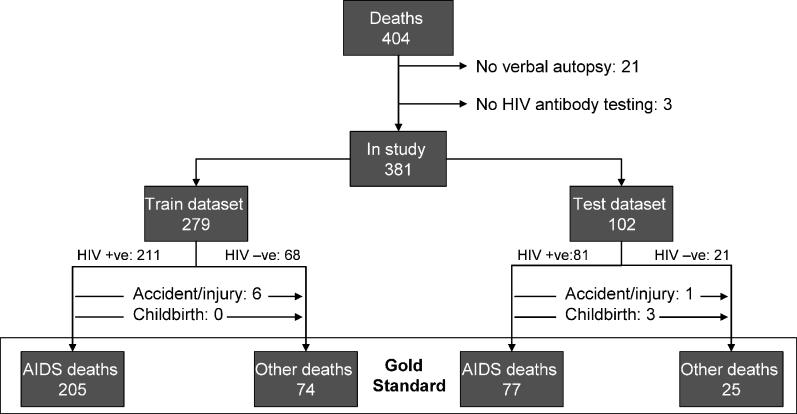
Derivation of Gold Standard from HIV Serostatus at Baseline in the Train and Test Datasets

A total of 404 deaths were recorded in the study period. Verbal autopsy was not performed on 21 of these deaths and HIV status at baseline was missing from another two deaths, leaving 381 complete records (94%) from the total population that died (Figure 1). Of this total, 292 (77%) were HIV positive at baseline. Accidents or injury were the cause of death in 18 patients, seven of whom were HIV-positive and 11 of whom were HIV-negative at baseline. As would be expected, given the higher death rate of those HIV infected the proportion of those dying of non-HIV-associated causes was less in the HIV positives. The odds of accident or injury death were lower in HIV-positive people (odds ratio [OR] = 0.17; 95% confidence interval [CI], 0.07–0.46). Direct obstetric causes accounted for the deaths of six women, three of whom were HIV-positive at baseline. The odds of direct obstetric death were lower in HIV-positive women (OR = 0.30; 95% CI, 0.06–1.5).

Classifying all HIV-positive non-accident/injury and non-childbirth deaths as “AIDS deaths” and all others as “non-AIDS deaths” gave a gold standard of 282 AIDS deaths (74%) and 99 Non-AIDS deaths (26%).

Of the 381 deaths studied, 279 (71%) were randomly assigned to the train dataset and 102 (29%) to the test dataset. In the train dataset, 73% of the deaths were AIDS-associated compared with 75% in the test dataset (χ^2^ *p* = 0.44).

### Construction of the Algorithm

Table 1 describes the signs and symptoms that showed an association with AIDS-deaths (LR ≥ 1.92, *p* ≤ 0.05). (The full table of investigated signs and symptoms can be found in Table S1.)

**Table 1:**
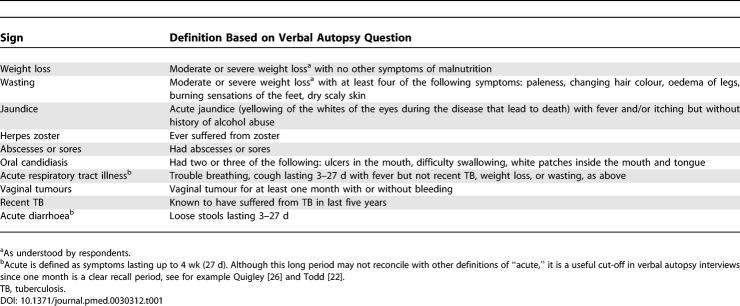
Signs and Symptoms Predictive of AIDS-Associated Deaths

**Figure 2:**
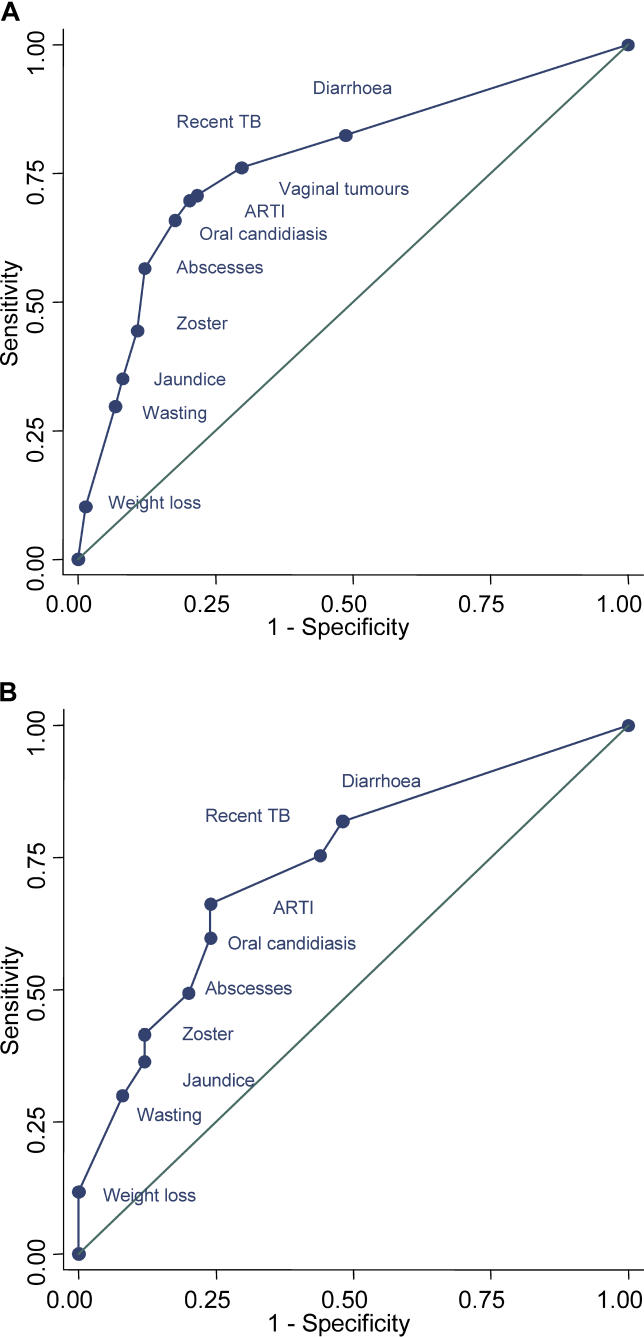
Modified Receiver Operator Characteristic Curves: Sensitivity Plotted against (1 − Specificity) Datasets are train (A) and test (B). In the test dataset, no individuals were reclassified as AIDS deaths because of presence of vaginal tumours; therefore no data point is plotted for that criterion in (B).

Table 2 shows the order in which criteria were added to the algorithm and the resulting sensitivity, specificity, predictive values, and post-test probabilities. Classifying all deaths as HIV-associated where one or more of the first eight criteria (weight loss, wasting, jaundice, herpes zoster, presence of abscesses or sores, oral candidiasis, acute respiratory tract infections, and vaginal tumours) were present gave a sensitivity of 71% (95% CI, 65%–77%) and a specificity of 78% (95% CI, 69%–88%) on the train dataset, correctly classifying 73% of deaths in the train dataset. Adding a ninth criteria (tuberculosis) resulted in a drop in specificity that was greater than the gain in sensitivity, as shown in the ROC curve in Figure 2. Thus the algorithm that included eight criteria optimised the trade-off between sensitivity and specificity.

**Table 2:**
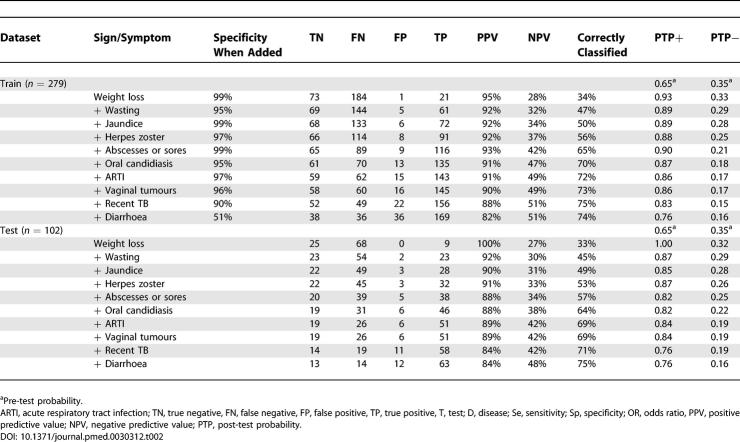
Sensitivity, Specificity, Likelihood Ratio, and Ranks Based on Likelihood Ratio, Additive Specificity, and Iterative Specificity of Selected Signs and Symptoms

Applying these eight criteria to the test dataset gave a sensitivity of 66% (95% CI, 0.56–0.77) and specificity of 76% (95% CI, 0.59–0.93).

No statistically significant associations were found in logistic regression models between correct classification and interviewer (LR test *p* = 0.13), time between death, and VA (*p* = 0.5), sex of the respondent (*p* = 0.6), side of family of respondent (*p* = 0.5), relationship of respondent to the deceased (*p* = 0.5), whether the respondent was the primary caregiver (*p* = 0.4), or sex of the deceased (*p* = 0.6). The proportion correctly classified was between 0.65 and 0.81 for all strata (Table 3).

**Table 3:**
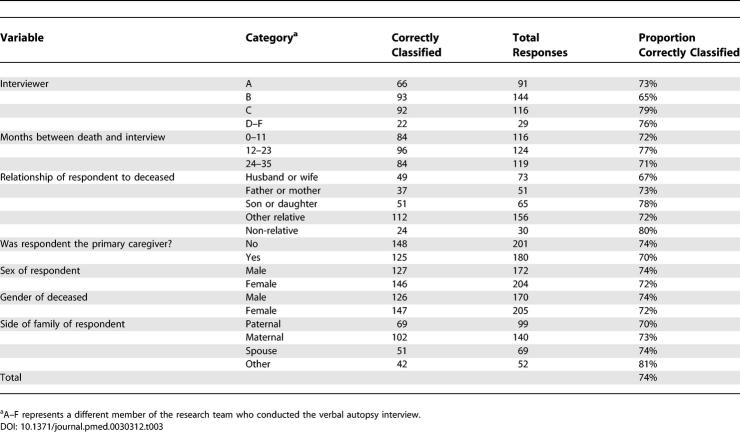
Proportion of Deaths Correctly Classified as AIDS or Non-AIDS Stratified by Characteristics of the Deceased, the Respondent, and Timing of the VA Relative to the Death

### Potential Misclassification in the Gold Standard

Given an underlying hazard of death of 7.45 per 1,000 person-years in this population [17], we would expect 30.6 deaths to occur in the HIV-positive population that were *not* a result of AIDS. Ten of these deaths would have been correctly reclassified because they were the result of observably non-AIDS causes (obstetric or accident/injury). Therefore, we estimate 5.4% (20.6/381) of all deaths or 7.2% (20.6/283) of HIV-positive deaths were misclassified in the gold standard.

### Prediction Based on the Algorithm

The positive predictive value using the eight criteria was 89% on the test dataset (Table 2). Because the algorithm was constructed on specificity rather than sensitivity, the positive predictive value exceeded the corresponding negative predictive value of 42%. Assuming a pre-test (prior) probability of 0.65 that a death was caused by AIDS, we calculate a post-test probability (+) of 0.84 if the death met one of the eight criteria of an AIDS death; the post-test probability (−) when none of the eight criteria were present was 0.19.

## Discussion

Analysis of verbal autopsy data in this rural Zimbabwean population revealed a distinct pattern of signs and symptoms associated with AIDS deaths. Using an algorithm built on specificity that minimised false positives, an overall sensitivity of 66% and a specificity of 76% for identifying AIDS deaths was achieved, and this method gave similar results when tested on a random sample of the data. These eight criteria were selected because they maximised the trade-off between sensitivity and specificity, while being independent of diagnosed TB.

Differential diagnosis of AIDS and TB is difficult amongst the living; it is not surprising therefore that classification using verbal autopsy is problematic. TB and other opportunistic infections of HIV infected people share many symptoms such as chronic cough, wasting and prolonged fever. In Zimbabwe, an estimated 67% of all new adult TB cases are HIV-positive and 7.5% of all AIDS deaths are due to TB [18]. One validated approach used in adult verbal autopsy study is to classify deaths as “Tuberculosis/AIDS” [19]. Our VA algorithm, which classifies AIDS independently from diagnosed TB is suited to quantifying the direct impact AIDS mortality in the community.

A classification system with known sensitivity and specificity has practical applications. First, the unbiased (UB) proportion of deaths that are AIDS-associated can be calculated as follows:

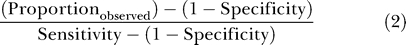



If we apply the observed proportion (designated P here) from the test dataset (P_true positive_ + P_false positive_ = 0.56), we would calculate a true proportion of AIDS deaths of 0.76 (95% CI, 0.70–0.81, calculated using the delta method) [20]. The real proportion from the gold standard was 0.74.

Furthermore, if we know the mortality rate ratio of HIV-positive individuals with respect to the HIV-negative population, the HIV prevalence can be “back-calculated” by the formula for proportion_UB_ below:

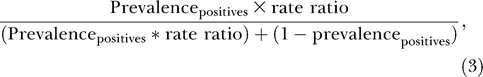
which can be rearranged to solve for prevalence_positive_:

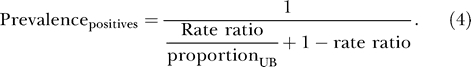



Based on data from the Manicaland cohort study (rate_positives_ = 0.071/y; rate_negatives_ = 0.0075/y; or rate ratio = 9.4) (note: similar mortality rate ratios have been observed in other African populations with different levels of prevalence [16,17,21–24]), we can estimate a population prevalence of 24.3% (95% CI, 18.0%–30.5%, calculated using the delta method [Protocol S2]). The observed prevalence, which was 23.0% at baseline [25], is close to the middle of this range. However, this calculation is presented only as an example of the method since here the same data source is used to estimate the input parameters (mortality rates and true proportion of deaths) as is used for the prevalence estimate. The accuracy of this estimation would need to be tested on other populations, but the potential to estimate HIV prevalence solely based on presence of signs/symptoms surrounding death is appealing, and has potential applications in demographic surveillance systems that perform VA but do not test for HIV. Dataset S1 contains a spreadsheet to calculate proportion_UB_, prevalence_positives_, and the associated confidence intervals.

Both the sensitivity and specificity of this tool may be considered modest. However, as the extrapolations presented above demonstrate, for many purposes it is more important that the characteristics of a diagnostic be *known* than that they be exceptionally precise and accurate.

Other methods of creating the classification system were investigated; for the sake of brevity these data are not shown. Preliminary analysis showed that these methods (LRs [similar to above], specificity [not stepwise], and multivariable odds ratio scoring as described by Quigley et al. [26]) were less accurate in correctly classifying deaths as AIDS-associated.

Using different approaches, others have also used the VA to identify AIDS deaths. Using a physician-based, rather than algorithm-based, approach, Kamali et al. [27] achieved 92% specificity and 92% positive predictive value of verbal autopsy to diagnose HIV-related adult deaths in Uganda. This highly valuable early study did not aim to devise simple criteria, used HIV serostatus as its gold standard, and was undertaken at a time of lower AIDS mortality. Combining a physician and algorithm-based VA, Urassa et al. [16] estimated that approximately half of all deaths in a population in rural Tanzania were caused by HIV/AIDS, although the VA was not validated within the context of their study. In addition, in Rakai District, Uganda, Sewankambo demonstrated the strong association of certain symptoms with mortality in HIV-positive people, although no individual symptom had a sensitivity greater than 40%, including the WHO definition of AIDS at 18% [23]. We speculate that the main reason our algorithm's results differed from the above studies stems from our use of a robust gold standard that applied to all deaths in the community, rather than only those in hospital records. Le Coeur et al. [28] also achieved comparatively high sensitivity and specificity by using a definition that included HIV serostatus in their reference standard.

The WHO definition of AIDS (two or more major signs plus one or more minor signs) was not a very accurate tool, classifying less than half of all deaths as a result of AIDS [29]. However, the predictors of AIDS death that we identified are largely consistent with the WHO case definition for AIDS surveillance [29]. Weight loss, pruritic dermatitis, herpes zoster, oropharangeal candidiasis, pneumonia (expanded definition), and invasive cervical cancer (expanded definition) were common to both, but more general elements of the WHO definition (prolonged fever and chronic diarrhoea) were not included in this algorithm.

How predictive would these eight signs and symptoms for AIDS mortality be in other settings? In this cohort, the post-test probability (if a death was associated with one of the eight criteria) was 84%. If none of the criteria were met there was a 19% chance that the death was AIDS-associated. However, these probabilities are affected by HIV prevalence and presence of differential symptoms in non-AIDS deaths. Thus, predictive power will be diminished in populations with higher background levels of these signs/symptoms (e.g., hepatitis); however, we do not expect the signs/symptoms used in this algorithm to be common in populations with low levels of AIDS mortality.

Studies of diagnostic validity face the problem of finding a suitable reference standard, and the major verbal autopsy validation studies have used hospital records [19,30]. In this way the VA is calibrated to attribute the same causes of death as those recorded in hospital settings, which is the source of many global mortality statistics, such as the ICD-10. One other comparatively small study in rural South Africa (*n* = 109) by Hosegood et al. [31] did in fact achieve somewhat higher sensitivity and specificity for AIDS mortality by physician review, using hospital records as a reference standard. However, there are two main problems with this approach that our study may have partially overcome. First, the populations who die in hospital in settings with poor health infrastructure may not be representative in terms of age, gender, or cause of death. In this study 25% of deaths amongst HIV-positive individuals occurred in hospital compared with 33% in HIV-negative persons (unpublished data). Second, in places where stigma and shame are borne by relatives of a person with AIDS, hospital records may not reflect the true number of AIDS deaths. Indeed, amongst the deaths in this study, only 71 (18%) had a death certificate, of which 31 (62%) of HIV-positive deaths noted AIDS/HIV. Therefore, for attribution to the single cause of AIDS, where it is known that mortality rates are approximately ten times higher amongst HIV-positive people, it is likely that fewer misclassifications would be made based principally on diagnostic results from up to three years prior to death as compared with hospital records. We estimate 5% misclassification of AIDS/non-AIDS deaths. Although any misclassification is not ideal in a gold standard, we argue that this level of misclassification compares favourably with hospital records in this setting and, importantly, provides a quantification of the level of misclassification which is impossible in most hospital-based studies. In the worst-case scenario, where all misclassifications in the gold standard are misclassified by the algorithm, we would expect a drop in the sensitivity to about 71% (down from 76%) and specificity to about 62% (down from 66%).

Incorrect classification of HIV status at baseline would affect the validity of the gold standard. However, the probability of misclassification due to incorrect testing status is very low, given the 99.6% sensitivity and specificity of the HIV dipstick test [14]. Likewise, the probability of seroconversion, progression to AIDS, and death within the follow-up period of three years is vanishingly small [21,32]. Although examination of the level of missing data and the proportion correctly classified by interview and respondent characteristics revealed no clear limitations, the problems of recall and quality of responses may remain difficult to quantify or detect. Longer recall has not been associated with lower sensitivity or specificity in validation studies, although work is needed to define the optimal and acceptable recall period [33].

Verbal autopsy is potentially an important way in which population levels and trends in AIDS-specific mortality can be monitored. The data can be collected as part of developing vital registration systems, sample vital registration systems [34], and local demographic surveillance systems. The data-derived set of criteria based on the history of eight signs and symptoms can be applied for surveillance of AIDS-related mortality in sub-Saharan Africa and other countries with generalized AIDS epidemics. These criteria should be further validated in time, with future data from this cohort, and place, through application to other settings in the region.

## Supporting Information

Dataset S1 Spreadsheet for Calculating Prevalence of AIDS Deaths and HIV (24 KB XLS)

Protocol S1 Verbal Autopsy Questionnaire (148 KB PDF)

Protocol S2 Statistical Appendix (18 KB DOC)

Table S1 Table of All Signs and Symptoms and Their Relationships with AIDS Deaths Abbreviations: D, disease; FN, false negative; FP, false positive; LR+, likelihood ratio when sign/symptom is present; Se, sensitivity; Sp, specificity; T, test; TN, true negative; TP, true positive; uOR, unadjusted odds ratio.
(198 KB DOC)

## Abbreviations

ARV: antiretroviral therapy
CI: confidence interval
LR: likelihood ratio
OR: odds ratio
ROC: receiver operator characteristic
UB: unbiased
VA: verbal autopsy
