# Supplementary material for: Creating and Validating an Algorithm to Measure AIDS Mortality in the Adult Population using Verbal Autopsy
Source: PLoS Med. 2006 Aug 8;3(8):e312. doi: 10.1371/journal.pmed.0030312 (PMC1526767; doi:10.1371/journal.pmed.0030312)
Supplement: Supplementary file 1 [file pmed.0030312.sd002.pdf]

REF.	QUESTIONS & FILTERS	CODING CATEGORIES			SKIP TO
**Q501**	**Ask the respondent for his/her account of the cause of death.**				
**Q502**	***During the two weeks before (NAME) died, did he/she suffer from any major injury, poisoning, burn or drowning?***	**Poisoning**	1		
		**Fall**	2		
		**Burn**	3		
		**Drowning**	4		
		**Alcohol intoxication**	5		
		**Ate toxic herbs/plants**	6		
		**Motor vehicle accident**	7		
		**Other injury**	8		
		**Death not due to injury**	9		**- Q504**
**Q503**	***Was it an accident, was it inflicted deliberately by someone else, or was the death self-inflicted?***	**Accident**	1		
		**Homicide**	2		
		**Suicide**	3		
		**Don't know**	98		
**Q504**	**Record whether deceased was male or female.**	**Male**	1		**- Q701**
		**Female**	2		

REF.	QUESTIONS & FILTERS	CODING CATEGORIES		SKIP TO
Q601	*How many children had (NAME) given birth to when she died?* **Do NOT include the last birth.**	Live births<br>Don't know	 98	
Q602	*Did (NAME) die during pregnancy or childbirth or within 6 weeks of giving birth?*	Yes<br>No<br>Don't know	1<br>2<br>98	- Q608
Q603	*Did (NAME) have her periods coming regularly?*	Yes<br>No<br>Don't know	1<br>2<br>98	
Q604	*Did (NAME) have a swelling growing out of the vagina?*	Yes<br>No<br>Don't know	1<br>2<br>98	 - Q606<br>- Q606
Q605	*For how long had this swelling been present?*	Months/years (mths / yrs)<br>Don't know	 98	
Q606	*Did (NAME) have bleeding from the vagina?*	Yes<br>No<br>Don't know	1<br>2<br>98	- Q701
Q607	*How long ago did she last have her period?*	Months/years (mths / yrs)<br>Don't know	 98	- Q609<br>- Q609
Q608	*How many months was she pregnant when she died?*	Month (mths)<br>Don't know	 98	
Q609	*Did she suffer from any complaints during her last pregnancy?*	Yes (specify)<br>No<br>Don't know	1<br>2<br>98	
Q610	*Did she attend antenatal clinics during her last pregnancy?*	Yes<br>No<br>Don't know	1<br>2<br>98	
Q611	*Did (NAME) have high blood pressure during pregnancy?*	Yes<br>No<br>Don't know	1<br>2<br>98	
Q612a	*Was she complaining of severe headaches?*	Yes<br>No<br>Don't know	1<br>2<br>98	
Q612b	*Was there bleeding during pregnancy?*	Yes<br>No<br>Don't know	1<br>2<br>98	
Q613	*Did (NAME) have oedema of the limbs during pregnancy?*	Yes<br>No<br>Don't know	1<br>2<br>98	
Q614	*Did (NAME) have malaria during pregnancy?*	Yes<br>No<br>Don't know	1<br>2<br>98	

REF.	QUESTIONS & FILTERS	CODING CATEGORIES		SKIP TO
**Q615**	***At what stage of the pregnancy did (NAME) die?***	**During delivery**	1	
		**Shortly before delivery**	2	
		**Well before delivery**	98	**- Q701**
**Q616**	***Was there excessive bleeding during delivery?***	**Yes**	1	
		**No**	2	
		**Don't know**	98	
**Q617**	***Was she complaining of severe headaches during delivery?***	**Yes**	1	
		**No**	2	
		**Don't know**	98	
**Q618**	***Did she have terrible abdominal pains during delivery that suddenly stopped before she died?***	**Yes**	1	
		**No**	2	
		**Don't know**	98	
**Q619**	***Did the placenta come out within half an hour of the birth of the child?***	**Yes**	1	
		**No**	2	
		**Don't know**	98	
**Q620**	***Did (NAME) have convulsions during delivery?***	**Yes**	1	
		**No**	2	
		**Don't know**	98	
**Q621**	***Was there high fever starting after delivery?***	**Yes**	1	
		**No**	2	**- Q623**
		**Don't know**	98	**- Q623**
**Q622**	***Did it start immediately after delivery or after a few days?***	**Immediately**	1	
		**After a few days**	2	
		**Don't know**	98	
**Q623**	***Where did the delivery take place?***	**Home**	1	
		**Relative's home**	2	
		**TBA's house**	3	
		**Provincial hospital**	4	
		**District hospital**	5	
		**Other local hospital**	6	
		**Clinic**	7	
		**Other (specify)**	8	
		**Don't know**	98	
**Q624**	***Who was in attendance at the birth?***	**Doctor**	1	
		**Nurse**	2	
		**Midwife**	3	
		**TBA**	4	
		**Don't know**	98	
**Q625**	***Is the child still alive?***	**Yes**	1	
		**Stillbirth**	2	
		**Died after birth**	3	
		**Don't know**	98	

REF.	QUESTIONS & FILTERS	CODING CATEGORIES		SKIP TO
Q701	*For how long had (NAME) been ill before he/she died?*	days / mths / yrs		
		Don't know	98	
Q702	*Did (NAME) have frequent loose stools or liquid stools during the disease that led to death?*	Yes	1	
		No	2	- Q710
		Don't know	98	- Q710
Q703	*How many stools did he/she have in a day?*	Number of stools		
		Don't know	98	
Q704	*How long did the diarrhoea last?*	days / mths / yrs		
		Don't know	98	
Q705	*Did (NAME) have blood in the stools?*	Yes	1	
		No	2	- Q708
		Don't know	98	- Q708
Q706	*For how long did he/she have blood in the stools?*	days / mths / yrs		
		Don't know	98	
Q707	*Did the stools look like rice water (whitish)?*	Yes	1	
		No	2	
		Don't know	98	
Q708	*Did the eyes become more sunken?*	Yes	1	
		No	2	
		Don't know	98	
Q709	*Did he/she suffer from dehydration?*	Yes	1	
		No	2	
		Don't know	98	
Q710	*Did (NAME) have a cough?*	Yes	1	
		No	2	- Q716
		Don't know	98	- Q716
Q711	*For how long did this last?*	days / mths / yrs		
		Don't know	98	
Q712	*Did (NAME) cough sputum?*	Yes	1	
		No	2	
		Don't know	98	
Q713	*Did (NAME) have severe pain while coughing?*	Yes	1	
		No	2	
		Don't know	98	
Q714	*Did (NAME) cough blood?*	Yes	1	
		No	2	
		Don't know	98	
Q715	*Did (NAME) cough more at night than in the morning?*	Yes	1	
		No	2	
		Don't know	98	

REF.	QUESTIONS & FILTERS	CODING CATEGORIES		SKIP TO
Q716	*Did (NAME) have trouble breathing during the illness that led to death?*	Yes<br>No<br>Don't know	1<br>2<br>98	 - Q721<br>- Q721
Q717	*For how long did this last?*	days / mths / yrs<br>Don't know	 98	
Q718	*Was (NAME) unable to lie down flat in bed because of shortness of breath?*	Yes<br>No<br>Don't know	1<br>2<br>98	
Q719	*During the past years did (NAME) have attacks of shortness of breath and noisy breathing (asthma)?*	Yes<br>No<br>Don't know	1<br>2<br>98	
Q720	*During the past year, was (NAME) short of breath upon exercise?*	Yes<br>No<br>Don't know	1<br>2<br>98	
Q721	*Did (NAME) have pneumonia?*	Yes<br>No<br>Don't know	1<br>2<br>98	
Q722	*How long ago is it since (NAME) suffered from tuberculosis?*	mths / yrs<br>Never<br>Don't know	 97<br>98	
Q723	*Did (NAME) have profuse night sweating?*	Yes<br>No<br>Don't know	1<br>2<br>98	
Q724	*Did (NAME) have a fever?*	Yes<br>No<br>Don't know	1<br>2<br>98	 - Q728<br>- Q728
Q725	*For how long did this last?*	days / mths / yrs<br>Don't know	 98	
Q726	*Was the fever present all the time or intermittent?*	Present all the time<br>Intermittent<br>Don't know	1<br>2<br>98	
Q727	*Was (NAME) shivering before having fever?*	Yes<br>No<br>Don't know	1<br>2<br>98	
Q728	*During the illness that led to death was (NAME) unconscious or very confused?*	Yes<br>No<br>Don't know	1<br>2<br>98	 - Q730<br>- Q730
Q729	*For how long did this last?*	days / mths / yrs<br>Don't know	 98	
Q730	*During the illness that led to death, did (NAME) have convulsions?*	Yes<br>No<br>Don't know	1<br>2<br>98	

REF.	QUESTIONS & FILTERS	CODING CATEGORIES		SKIP TO
**Q731**	***During the illness that led to death, did (NAME) have neck stiffness?***	**Yes**<br>**No**<br>**Don't know**	1<br>2<br>98	
**Q732**	***During the illness that led to death, did (NAME) have severe headache?***	**Yes**<br>**No**<br>**Don't know**	1<br>2<br>98	
**Q733**	***During the illness that led to death, did (NAME) have problems opening his/her mouth?***	**Yes**<br>**No**<br>**Don't know**	1<br>2<br>98	
**Q734**	***During the illness that led to death, did (NAME) have spasms? (body muscles becoming very stiff)***	**Yes**<br>**No**<br>**Don't know**	1<br>2<br>98	
**Q735**	***Did (NAME) get a wound (e.g.: bed sores) during the last two weeks before death?***	**Yes**<br>**No**<br>**Don't know**	1<br>2<br>98	
**Q736**	***Was (NAME) unable to speak?***	**Yes**<br>**No**<br>**Don't know**	1<br>2<br>98	
**Q737**	***During the disease that led to death, did (NAME) loose weight?***	**Yes**<br>**No**<br>**Don't know**	1<br>2<br>98	 **- Q739**<br>**- Q739**
**Q738**	***Was the weight loss severe or moderate?***	**Severe**<br>**Moderate**<br>**Don't know**	1<br>2<br>98	
**Q739**	***During the disease that led to death, did (NAME) become very pale?***	**Yes**<br>**No**<br>**Don't know**	1<br>2<br>98	
**Q740**	***During the disease that led to death, did (NAME) suffer a yellowing of the whites of the eyes (jaundice)?***	**Yes**<br>**No**<br>**Don't know**	1<br>2<br>98	
**Q741**	***During the disease that led to death, did (NAME) have swollen legs?***	**Yes**<br>**No**<br>**Don't know**	1<br>2<br>98	
**Q742**	***Did the colour of his/her hair change?***	**Yes**<br>**No**<br>**Don't know**	1<br>2<br>98	
**Q743**	***Did (NAME) complain of burning sensations of the legs?***	**Yes**<br>**No**<br>**Don't know**	1<br>2<br>98	
**Q744**	***Did (NAME) have any skin problems during the disease that led to death?***	**Yes**<br>**No**<br>**Don't know**	1<br>2<br>98	 **- Q749**<br>**- Q749**
**Q745**	***For how many days did it last?***	**Days**<br>**Don't know**	 98	
**Q746**	***Where was the rash located?***	**All over the body**<br>**On specific parts only (specify)**<br>**Don't know**	1<br>2<br>98	

REF.	QUESTIONS & FILTERS	CODING CATEGORIES		SKIP TO
**Q747**	***Did (NAME) complain of itching of the skin?***	**Yes**<br>**No**<br>**Don't know**	1<br>2<br>98	
**Q748**	***Did the skin become very dry or scaly?***	**Yes**<br>**No**<br>**Don't know**	1<br>2<br>98	
**Q749**	***Did (NAME) have one localised dark swelling of skin?***	**Yes**<br>**No**<br>**Don't know**	1<br>2<br>98	
**Q750**	***Did (NAME) have abcesses or sores?***	**Yes**<br>**No**<br>**Don't know**	1<br>2<br>98	 **- Q752**<br>**- Q752**
**Q751**	***How many abscesses or sores?***	**One**<br>**Two to four**<br>**At least five**<br>**Don't know**	1<br>2<br>3<br>98	
**Q752**	***Has (NAME) ever had herpes zoster?***	**Yes**<br>**No**<br>**Don't know**	1<br>2<br>98	 **- Q754**<br>**- Q754**
**Q753**	***How many times?***	**Once**<br>**More than once**<br>**Don't know**	1<br>2<br>98	
**Q754**	***Did (NAME) have swellings?***	**Yes**<br>**No**<br>**Don't know**	1<br>2<br>98	 **- Q756**<br>**- Q756**
**Q755**	***Which parts were swollen?***<br><br>***Any other parts?***<br><br><br>**Probe for other parts.**	**Whole body swollen**<br>**Bumps all over body**<br>**Neck**<br>**Face**<br>**Feet, lower legs**<br>**Axilla (arm pit)**<br>**Groin**<br>**Abdomen**<br>**Other parts (specify)**<br>**Don't know**	1<br>2<br>3<br>4<br>5<br>6<br>7<br>8<br>9<br>98	
**Q756**	***Did (NAME) have protruded eyes?***	**Yes**<br>**No**<br>**Don't know**	1<br>2<br>98	
**Q757**	***Was (NAME) able to see well?***	**Yes**<br>**No**<br>**Don't know**	1<br>2<br>98	**- Q759**
**Q758**	***Was (NAME) able to see well when he/she was a child?***	**Yes**<br>**No**<br>**Don't know**	1<br>2<br>98	
**Q759**	***Was (NAME) known to have a heart problem?***	**Yes**<br>**No**<br>**Don't know**	1<br>2<br>98	

REF.	QUESTIONS & FILTERS	CODING CATEGORIES		SKIP TO
Q760	*Was (NAME) known to have high blood pressure?*	Yes<br>No<br>Don't know	1<br>2<br>98	
Q761	*Was (NAME) known to have diabetes?*	Yes<br>No<br>Don't know	1<br>2<br>98	
Q762	*Was (NAME) known to have HIV infection?*	Yes<br>No<br>Don't know	1<br>2<br>98	
Q763	*Did (NAME) have "sickle cell"?*	Yes<br>No<br>Don't know	1<br>2<br>98	
Q764	*Was (NAME) healthy as a child?*	Yes<br>No<br>Don't know	1<br>2<br>98	- Q768
Q765	*Did (NAME) have attacks of severe joint pains during his/her life?*	Yes<br>No<br>Don't know	1<br>2<br>98	
Q766	*Did (NAME) have attacks of becoming yellow during his/her lifetime?*	Yes<br>No<br>Don't know	1<br>2<br>98	
Q767	*Are there other family members with a similar disease?*	Yes<br>No<br>Don't know	1<br>2<br>98	
Q768	*Did (NAME) have ulcers in the mouth?*	Yes<br>No<br>Don't know	1<br>2<br>98	
Q769	*Did (NAME) have difficulty swallowing?*	Yes<br>No<br>Don't know	1<br>2<br>98	
Q770	*Did (NAME) have white patches on the inside of the mouth and tongue?*	Yes<br>No<br>Don't know	1<br>2<br>98	
Q771	*Did(NAME) suffer from vomitting?*	Yes<br>No<br>Don't know	1<br>2<br>98	 - Q773<br>- Q773
Q772	*Did (NAME) vomit blood?*	Yes<br>No<br>Don't know	1<br>2<br>98	
Q773	*Did (NAME) have severe pains in the abdomen?*	Yes<br>No<br>Don't know	1<br>2<br>98	- Q776
Q774	*Did (NAME) dislike certain foods?*	Yes<br>No<br>Don't know	1<br>2<br>98	 - Q776<br>- Q776
Q775	*Which foods did he/she dislike?*	Beans<br>Peppers<br>Other (specify)	1<br>2<br>98	

REF.	QUESTIONS & FILTERS	CODING CATEGORIES		SKIP TO
**Q776**	***Did (NAME) experience any problems/changes in urination?***	**Yes**	1	
		**No**	2	**- Q782**
		**Don't know**	98	**- Q782**
**Q777**	***Did (NAME) have pain during urination?***	**Yes**	1	
		**No**	2	
		**Don't know**	98	
**Q778**	***During the illness that led to death, did (NAME) pass brown or dark urine?***	**Yes**	1	
		**No**	2	
		**Don't know**	98	
**Q779**	***During the illness that led to death, did (NAME) have blood in the urine?***	**Yes**	1	
		**No**	2	
		**Don't know**	98	
**Q780**	***Was (NAME) unable to pass urine during the last days before death?***	**Yes**	1	
		**No**	2	
		**Don't know**	98	
**Q781**	***Did (NAME) have to urinate a lot?***	**Yes**	1	
		**No**	2	
		**Don't know**	98	
**Q782**	***Did (NAME) have unusually excessive thirst?***	**Yes**	1	
		**No**	2	
		**Don't know**	98	
**Q783**	***Did (NAME) complain of severe body pains?***	**Yes**	1	
		**No**	2	**- Q785**
		**Don't know**	98	**- Q785**
**Q784**	***Which parts was (NAME) complaining of?***	**Whole body**	1	
		**Abdomen**	2	
		**Limbs**	3	
	<u>**Probe for any other parts.**</u>	**Chest**	4	
		**Head**	5	
		**Bones**	6	
		**Other parts (specify)**	8	
		**Don't know**	98	
**Q785**	***Did (NAME) have allergic skin reactions to drugs?***	**Yes**	1	
		**No**	2	
		**Don't know**	98	
**Q786**	***Was(NAME) unable to move limbs? (paralysis)?***	**Yes: one sided**	1	
		**Yes: both legs**	2	
		**Yes: both arms**	3	
	If yes, which ones?	**No**	4	
		**Don't know**	98	
**Q787**	***During his/her lifetime, did (NAME) usually drink a lot of alcohol?***	**Yes**	1	
		**No**	2	
		**Don't know**	98	
**Q788**	***Does (NAME) have a spouse who is unwell?***	**No**	1	
		**Yes: acutely ill**	2	
		**Yes: chronically ill**	3	
		**Don't know**	98	

REF.	QUESTIONS & FILTERS	CODING CATEGORIES		SKIP TO
Q789	*During the disease that led to death, was advice or treatment sought from anywhere / anyone?*<br><br>Record all mentioned.	Nobody	1	
		Relative/friends	2	
		N'anga	3	
		Faith healer	4	
		Pharmacist	5	
		Private health facility	6	
		Government dispensary / clinic	7	
		Hospital	8	
		Don't know	98	
Q790	*Was he/she given anything when he/she was ill?*	Yes	1	
		No	2	- Q792
		Don't know	98	- Q792
Q791	*What treatment was given?*<br><br>*Anything else?*<br><br>Record all mentioned.	Tablets	1	
		Capsules	2	
		Injections	3	
		ORS packet solution	4	
		Syrup	5	
		Home remedy	6	
		Traditional medicine	7	
		Other (specify)	8	
		Don't know	98	
Q792	*Where did (NAME) die?*	Hospital/clinic	1	
		On way to hospital	2	
		At home	3	
		Elsewhere	4	
		Don't know	98	
Q792	*Is there a death certificate?*	Yes		
		No		- End
		Don't know		- End
Q793	Check name.	Correct		
		Incorrect		
Q794	Record date of death per death certificate.	mnth yr		
Q795	Record place of death per death certificate.	Name of place. ________		
		Harare	1	
		Mutare	2	
		Rusape	3	
		Other town or city	4	
		Small town or growth point	5	
		Estate/mining area	6	
		Roadside business centre	7	
		Rural business centre	8	
		Communal/resettlement area	9	
		Not stated	98	
Q796	Record age at death per death certificate.	yrs		
Q797	Record cause of death per death certificate.	Immediate cause ________		
		Underlying cause ________		
