# Supplementary material for: Creating and Validating an Algorithm to Measure AIDS Mortality in the Adult Population using Verbal Autopsy
Source: PLoS Med. 2006 Aug 8;3(8):e312. doi: 10.1371/journal.pmed.0030312 (PMC1526767; doi:10.1371/journal.pmed.0030312)
Supplement: Supplementary file 2 [file pmed.0030312.sd003.doc]

**Appendix 2**

The following formulae were derived using the delta method to calculate variance of the (1) unbiased proportion and (2) HIV prevalence.

(1) Variance of unbiased proportion of deaths that were AIDS associated

$\text{Var}(P_{\text{ub}})=\left( \frac{P_{\text{obs}}-(1-\text{Sp})}{\text{Se}-(1-\text{Sp})} \right)^{2}\begin{aligned} \left\{ \frac{\text{Var}(P_{\text{obs}})+\text{Var}(\text{Sp})}{\left( P_{\text{obs}}-(1-\text{Sp}) \right)^{2}}-\ldots| \right\}\left\{ \frac{2\text{Var}(\text{Sp})}{(P_{\text{obs}}-(1-\text{Sp}))(\text{Se}-(1-\text{Sp}))}+\ldots| \right\} \end{aligned}\left\{ \right\}$

(2) Variance of the estimated HIV prevalence, assuming that the mortality rate ratio (RR) is independent of the proportion of deaths caused by HIV/AIDS

$$\text{Var}(\text{Pr}\text{ev}_{\text{pos}})=\begin{aligned} \left( \left( \frac{\text{RR}}{P_{\text{ub}}} \right)^{2}\left\{ \frac{\text{Var}(\text{RR})}{\text{RR}^{r}}+\frac{\text{Var}(P_{\text{ub}})}{(P_{\text{ub}})^{2}} \right\}+\text{Var}(\text{RR})-\ldots| \right) \\ \end{aligned}$$
