# Supplementary material for: Creating and Validating an Algorithm to Measure AIDS Mortality in the Adult Population using Verbal Autopsy
Source: PLoS Med. 2006 Aug 8;3(8):e312. doi: 10.1371/journal.pmed.0030312 (PMC1526767; doi:10.1371/journal.pmed.0030312)
Supplement: Supplementary file 3 [file pmed.0030312.st001.doc]

|  | **TN** | **FN** | **FP** | **TP** |  |  |  |  |
| --- | --- | --- | --- | --- | --- | --- | --- | --- |
|  | **T-D-** | **T-D+** | **T+D-** | **T+D+** | **Se** | **Sp** | **uOR** | **LR+** |
|  |  |  |  |  |  |  |  |  |
| **Gastrointestinal** |  |  |  |  |  |  |  |  |
| Acute diarrhoea (< 22 days) | 81 | 212 | 18 | 70 | 25% | 82% | 1.48 | 1.37 |
| Acute diarrhoea (< 22 days) without dehydration | 81 | 213 | 18 | 69 | 24% | 82% | 1.46 | 1.35 |
| Acute diarrhoea (< 22 days) with signs of dehydration (sunken eyes or reported dehydration) | 84 | 223 | 15 | 59 | 21% | 85% | 1.48 | 1.38 |
| Cholera: White stools, > 10 per day and no fever | 99 | 281 | 0 | 1 | 0% | 100% | … | … |
| Dysentery (> 1 day diarrhoea and blood in stool) | 97 | 272 | 2 | 10 |  |  | … |  |
| Chronic diarrhoea (> 21 days) | 76 | 171 | 23 | 111 | 39% | 77% | 1.78** | 1.69 |
| Any diarrhoea (>days) | 59 | 133 | 40 | 149 | 53% | 60% | 1.65* | 1.31 |
|  |  |  |  |  |  |  |  |  |
| **Respiratory** |  |  |  |  |  |  |  |  |
| Fever with cough > 2 days & difficulty breathing > 1 day | 85 | 193 | 14 | 89 | 32% | 86% | 2.80*** | 2.23 |
| Fever with cough > 2 days & painful breathing > 1 day | 85 | 198 | 14 | 84 | 30% | 86% | 2.57** | 2.11 |
| ARTI: Fever with cough > 2 days, no recent TB, weight loss or wasting | 93 | 247 | 6 | 35 | 12% | 94% | 2.19* | 2.05 |
| Chronic cough (> 27 days) | 66 | 127 | 33 | 155 | 55% | 67% | 2.44** | 1.65 |
| Reported TB in last year | 90 | 224 | 9 | 58 | 21% | 91% | 2.59** | 2.26 |
| Recent reported TB (last 5 years) | 85 | 199 | 14 | 83 | 29% | 86% | 2.53** | 2.08 |
| TB (symptoms) Chronic cough (> 20 days) with night sweats and weight loss | 79 | 178 | 20 | 104 | 37% | 80% | 2.30** | 1.83 |
| Shortness of breath from asthma lasting > 27 days | 99 | 282 | 0 | 0 | … | … | … |  |
| Extrapulmonary TB: swelling in the neck, fever & weight loss | 96 | 270 | 3 | 12 | 4% | 97% | 1.92 | 1.40 |
|  |  |  |  |  |  |  |  |  |
| **Fever/malaria/meningitis** |  |  |  |  |  |  |  |  |
| Fever > 1 day | 59 | 126 | 40 | 156 | 55% | 60% | 1.82** | 1.37 |
| Fever > 1 day and cold shivers | 80 | 192 | 19 | 90 | 32% | 81% | 2.00** | 1.66 |
| Fever > 1 day, cold shivers and paleness | 87 | 216 | 12 | 66 | 23% | 88% | 2.40** | 1.93 |
| Fever > 1 day, convulsions and coma | 84 | 225 | 15 | 57 | 20% | 85% |  | 1.33 |
| Meningitis: Fever > 1 day, stiff neck and severe headache | 95 | 270 | 4 | 12 | 4% | 96% | 1.05 | 1.05 |
| Sickle cell disease: History and severe body pains | 98 | 277 | 1 | 5 | 2% | 99% | 1.78 | 1.76 |
| Tetanus: Spasms and history of wound | 97 | 276 | 2 | 6 | 2% | 98% | 1.05 | 1.05 |
|  |  |  |  |  |  |  |  |  |
| **Cardiovascular accident** |  |  |  |  |  |  |  |  |
| Coma without fever | 93 | 265 | 6 | 17 | 6% | 94% | 0.99 | 0.99 |
| Paralysis without fever | 96 | 268 | 3 | 14 | 5% | 97% | 1.67 | 1.64 |
| Paralysis and inability to speak without fever | 99 | 276 | 0 | 6 | 2% | 100% | … | … |
|  |  |  |  |  |  |  |  |  |
| **Heart condition** |  |  |  |  |  |  |  |  |
| History of heart condition | 91 | 264 | 8 | 18 | 6% | 92% | 0.77 | 0.79 |
| History of heart condition and swollen legs | 98 | 281 | 1 | 1 | 0% | 99% | 0.55 | 0.35 |
| Unable to lie down flat, night coughing & tired upon exercise | 92 | 259 | 7 | 23 | 8% | 93% | 1.16 | 1.15 |
|  |  |  |  |  |  |  |  |  |
| **Weight loss and malnutrition** |  |  |  |  |  |  |  |  |
| Weight loss | 61 | 69 | 38 | 213 | 76% | 62% | 4.95*** | 1.97 |
| Weight loss with paleness | 73 | 141 | 26 | 141 | 50% | 74% | 2.80*** | 1.90 |
| Weight loss with changing hair colour | 96 | 260 | 3 | 22 | 8% | 97% | 2.09*** | 2.57 |
| Weight loss with oedema of the legs | 92 | 273 | 7 | 9 | 3% | 93% | 1.60* | 0.45 |
| Weight loss with burning sensations of the feet | 99 | 272 | 0 | 10 | 4% | 100% | 2.98*** | … |
| Weight loss with dry scaly skin | 98 | 281 | 1 | 1 | 0% | 99% | 2.09** | 0.35 |
| Weight loss with no other symptoms of malnutrition | 98 | 252 | 1 | 30 | 11% | 99% | 11.7** | 10.53 |
|  |  |  |  |  |  |  |  |  |
| **Signs of malnutrition (as above)** |  |  |  |  |  |  |  |  |
| 1 or more | 53 | 62 | 46 | 220 | 78% | 54% | 2.83*** | 1.68 |
| 2 or more | 66 | 139 | 33 | 143 | 51% | 67% | 1.51* | 1.52 |
| 3 or more | 84 | 190 | 15 | 92 | 33% | 85% | 2.03* | 2.15 |
| 4 or more | 94 | 228 | 5 | 54 | 19% | 95% | 3.69** | 3.79 |
| 5 | 97 | 262 | 2 | 20 | 7% | 98% | 3.70* | 3.51 |
|  |  |  |  |  |  |  |  |  |
| **Acute liver disease** |  |  |  |  |  |  |  |  |
| Jaundice | 92 | 249 | 7 | 33 | 12% | 93% | 1.74 | 1.66 |
| Jaundice with dark urine or pale stools | 98 | 280 | 1 | 2 | 1% | 99% | 0.70 | 0.70 |
| Jaundice with fever | 95 | 276 | 3 | 7 | 2% | 97% | 2.80 | 0.81 |
| Jaundice with itching | 98 | 275 | 1 | 7 | 2% | 99% | 1.71 | 2.46 |
| Jaundice with history of alcohol abuse | 97 | 269 | 2 | 13 | 5% | 98% | 1.18 | 2.28 |
| Acute jaundice with fever and/or itching but without history of alcohol abuse | 96 | 258 | 3 | 24 | 9% | 97% | 2.97* | 2.81 |
|  |  |  |  |  |  |  |  |  |
| **Chronic jaundice** |  |  |  |  |  |  |  |  |
| Long term history of jaundice | 98 | 280 | 1 | 2 | 1% | 99% | 0.70 | 0.70 |
| Long term history of jaundice with abdominal swelling | 99 | 282 | 0 | 0 | 0% | 100% | … | … |
| Long term history of jaundice with abdominal swelling | 99 | 282 | 0 | 0 | 0% | 100% | … | … |
| Long term history of jaundice with itching | 99 | 280 | 0 | 2 | 1% | 100% | … | … |
| Long term history of jaundice and alcohol abuse | 99 | 280 | 0 | 2 | 1% | 100% | … | … |
|  | 99 | 280 | 0 | 2 | 1% | 100% | … | … |
|  |  |  |  |  |  |  |  |  |
| **Diabetes** |  |  |  |  |  |  |  |  |
| History of diabetes | 96 | 277 | 3 | 5 | 2% | 97% | 0.58 | 0.59 |
| History of diabetes and vision problems after normal child vision | 97 | 281 | 2 | 1 | 0% | 98% | 0.17 | 0.18 |
| History of diabetes and frequent urination/thirst | 99 | 278 | 0 | 4 | 1% | 100% | 1.76 | … |
| History of diabetes and weight loss | 98 | 282 | 1 | 0 | 0% | 99% | 1.05 | 0.00 |
|  |  |  |  |  |  |  |  |  |
| **Renal disease** |  |  |  |  |  |  |  |  |
| Swollen body and anuria | 99 | 282 | 0 | 0 | 0% | 100% | … | … |
| Swollen face and anuria/polyuria | 99 | 282 | 0 | 0 | 0% | 100% | … | … |
|  |  |  |  |  |  |  |  |  |
| **Renal/ bladder cancer** |  |  |  |  |  |  |  |  |
| Painful urination and blood in urine | 96 | 282 | 3 | 0 | 0% | 97% | 0 | 0.00 |
| Painful urination and anuria with blood in urine | 97 | 282 | 2 | 0 | 0% | 98% | 0 | 0.00 |
|  |  |  |  |  |  |  |  |  |
| **Maternal** |  |  |  |  |  |  |  |  |
| Post partum death - puerperal sepsis (up to 42 days post-partum) | 49 | 152 | 1 | 2 | 1% | 98% | 0.70 | 0.65 |
| Indirect causes: hepatitis/suicide | 47 | 151 | 3 | 3 | 2% | 94% | 0.34 | 0.32 |
|  |  |  |  |  |  |  |  |  |
| **Vaginal tumours** |  |  |  |  |  |  |  |  |
| Vaginal bleeding | 35 | 78 | 15 | 76 | 49% | 70% | 2.07 | 1.65 |
| Vaginal tumour for at least one month | 49 | 148 | 1 | 6 | 4% | 98% | 2.10 | 1.95 |
| Vaginal bleeding and tumour for at least one month | 48 | 146 | 2 | 8 | 5% | 96% | 0.70 | 1.30 |
|  |  |  |  |  |  |  |  |  |
| **Other infections** |  |  |  |  |  |  |  |  |
| Ever had herpes zoster | 93 | 208 | 6 | 74 | 26% | 94% | 3.95** | 4.33 |
| Presence of abscesses or sores | 90 | 218 | 9 | 64 | 23% | 91% | 2.94** | 2.50 |
| Oral candidiasis: 2 or more of the following: ulcers in the mouth, difficulty swallowing, white patches inside the mouth and tongue | 90 | 204 | 9 | 78 | 28% | 91% | 3.82*** | 3.04 |
